# Enhancing fine-grained intra-urban dengue forecasting by integrating spatial interactions of human movements between urban regions

**DOI:** 10.1371/journal.pntd.0008924

**Published:** 2020-12-21

**Authors:** Kang Liu, Meng Zhang, Guikai Xi, Aiping Deng, Tie Song, Qinglan Li, Min Kang, Ling Yin

**Affiliations:** 1 Shenzhen Institutes of Advanced Technology, Chinese Academy of Sciences, Shenzhen, Guangdong, China; 2 Guangdong Provincial Center for Disease Control and Prevention, Guangzhou, Guangdong, China; 3 University of Chinese Academy of Sciences, Beijing, China; Institute for Disease Modeling, UNITED STATES

## Abstract

**Background:**

As a mosquito-borne infectious disease, dengue fever (DF) has spread through tropical and subtropical regions worldwide in recent decades. Dengue forecasting is essential for enhancing the effectiveness of preventive measures. Current studies have been primarily conducted at national, sub-national, and city levels, while an intra-urban dengue forecasting at a fine spatial resolution still remains a challenging feat. As viruses spread rapidly because of a highly dynamic population flow, integrating spatial interactions of human movements between regions would be potentially beneficial for intra-urban dengue forecasting.

**Methodology:**

In this study, a new framework for enhancing intra-urban dengue forecasting was developed by integrating the spatial interactions between urban regions. First, a graph-embedding technique called Node2Vec was employed to learn the embeddings (in the form of an *N*-dimensional real-valued vector) of the regions from their population flow network. As strongly interacting regions would have more similar embeddings, the embeddings can serve as “interaction features.” Then, the interaction features were combined with those commonly used features (e.g., temperature, rainfall, and population) to enhance the supervised learning–based dengue forecasting models at a fine-grained intra-urban scale.

**Results:**

The performance of forecasting models (i.e., SVM, LASSO, and ANN) integrated with and without interaction features was tested and compared on township-level dengue forecasting in Guangzhou, the most threatened sub-tropical city in China. Results showed that models using both common and interaction features can achieve better performance than that using common features alone.

**Conclusions:**

The proposed approach for incorporating spatial interactions of human movements using graph-embedding technique is effective, which can help enhance fine-grained intra-urban dengue forecasting.

## Author summary

Dengue fever, a mosquito-borne infectious disease, has become a serious public health problem in many tropical and subtropical regions worldwide, such as Southeast Asian countries and the Guangdong Province in China. In the absence of an effective vaccine at present, disease surveillance and mosquito control remain the primary means of controlling the spread of the disease. At an intra-urban setting, it is important to predict the spatial distribution of future patients, which can help government agencies to establish precise and targeted prevention measures beforehand. Considering the fast virus spread within a city because of a highly dynamic population flow, we proposed a novel approach to enhancing fine-grained intra-urban dengue forecasting by integrating spatial interactions of human movements between urban regions. First, using a graph-embedding model called Node2Vec, the embeddings of the regions were learned from their population interaction network so that strongly interacted regions would have more similar embeddings. Secondly, serving as interaction features, the embeddings were combined with the commonly used features as inputs of the forecasting models. The experimental results indicated that the performance of the models can be improved by incorporating the interaction features, confirming the effectiveness of our proposed strategy in enhancing fine-grained intra-urban dengue forecasting.

## Introduction

Dengue fever (DF) is a mosquito-borne infectious disease caused by the dengue virus (DENV) (four serotypes DENV-1, -2, -3, and -4), and has spread through tropical and subtropical regions worldwide in recent decades. It is transmitted by the *Aedes* mosquitoes and, in urban areas, primarily by the anthropophilic *Aedes aegypti* [1]. Globally, the total number of dengue infections has been estimated to be 390 million per year [2], the majority of which are in Asia [1,3,4]. In mainland China, DF cases have been reported every year since 1997; approximately 94% of local cases were reported from Guangdong Province, and 83% of these cases were in its provincial capital city, Guangzhou [5].

At present, there are no specific treatment and effective vaccines regimens for dengue infection; interrupting the pathogen transmission through mosquito control remains the most effective means of dengue control and prevention [1,6]. Related government departments in various regions (e.g., the Guangdong Provincial Center for Disease Control and Prevention and the National Environment Agency of Singapore) usually deploy community workers for eliminating the potential breeding grounds and call on residents to clean up stagnant water and kill mosquitos through various propaganda means [1].

Consequently, an accurate early warning of dengue epidemic is important for timely and targeted vector control and prevention. To achieve this, various models have been proposed for dengue forecasting, including autoregressive models [7–10], generalized linear models [11–13], Poisson regression models [14–16], Bayesian hierarchical models [17,18], machine learning models such as artificial neural network (ANN) and support vector machine (SVM) [19–20], and deep learning models such as long short-term memory (LSTM) [21–22]. For instance, a time series Poisson multivariate regression model, that allows warning 16 weeks in advance of dengue epidemics, was developed in Singapore [16]. Some studies demonstrated that the least absolute shrinkage and selection operator (LASSO) regressions achieved good performance in dengue forecasting at both the city and neighborhood levels, and have been deployed by the Environmental Health Institute of Singapore to guide vector control [1,23]. According to a dengue forecasting study conducted at both the city (i.e., cities in Guangdong Province) and provincial levels (i.e., five provinces in China), the SVM-based regression (with linear kernel) outperformed other frequently-used algorithms such as gradient boosted regression tree, negative binomial regression, LASSO, and generalized additive model [19]. Existing forecasting models have primarily relied on two types of predictors, i.e., temporal autocorrelation and an association with weather or climate [9,13,16,18,24]. Temporal autocorrelation results from the infectious nature of the dengue viruses wherein cases are more likely to appear in the near future when the current prevalence of infection is high, while weather or climate factors such as temperature, precipitation, and humidity are important determinants of mosquito reproduction, longevity, and virus transmission ability [9]. With the exclusion of past cases and meteorological variables, the population [14,16,23], Internet search index [12,10,19], street view images [25], and dengue-related phone calls from telephone triage services [20] have also been proven as useful predictors for dengue forecasting. In addition, the influence of human mobility [26–29], land use [30], road network [3,31], population structure [32], and urban village [4] on dengue transmission has also been investigated.

Despite the fact that several approaches have been developed for dengue forecasting, the majority have focused on temporal prediction at national [9,12,33], subnational [14,19,34], and city levels [10,13,17,24]. However, the risks may vary across a city because of the spatial heterogeneity of sociodemographic and environmental conditions within the city [1,35,36]. Consequently, dengue forecasting at a finer spatial resolution is necessary and of great importance for precise dengue control and prevention.

This study aimed to establish a fine-grained intra-urban dengue forecasting framework for identifying target areas with greater risk in the near future. Considering that highly dynamic population flows greatly facilitate the spread of virus within cities [26], integrating spatial interactions between urban regions would be useful for dengue forecasting. To achieve this goal, this study ingeniously introduced the graph-embedding technique to capture spatial interactions of human movements between urban regions. In particular, considering regions as nodes and population flows between the regions as edge weights, the graph-embedding model Node2Vec was applied to learn the embedding of each region from the population interaction network. Serving as interaction features, the embeddings were combined with other commonly used features as inputs to enhance the existing forecasting models such as SVM, LASSO, and ANN. The effectiveness of the proposed approach was validated on township-level dengue predictions in Guangzhou, China.

## Materials and methods

### Study area and data

#### Study area

Guangzhou is the capital city of Guangdong province in South China, serving as an international port and an important foreign trade gateway into China. As one of China’s four largest cities (i.e., Beijing, Shanghai, Guangzhou, and Shenzhen), Guangzhou has an area of 7434 km^2^ and about 14.90 million permanent residents (http://www.gz.gov.cn/xw/zwlb/bmdt/stjj/content/post_5523428.html). The climate of Guangzhou is humid and subtropical, with high temperatures and humidity in summer and is comparatively mild and dry in winter; the annual mean temperature and cumulative precipitation are about 22°C and 1,800 mm, respectively. Guangzhou is situated close to Southeast Asian countries (e.g., Thailand, Singapore, Malaysia, Laos, and Vietnam) where dengue has been hyperendemic for decades, posing a large disease burden [19]. Its suitable climate, large floating and foreign population, and close proximity to Southeast Asia render Guangzhou the most dengue-threatened city in China.

**Fig 1:**
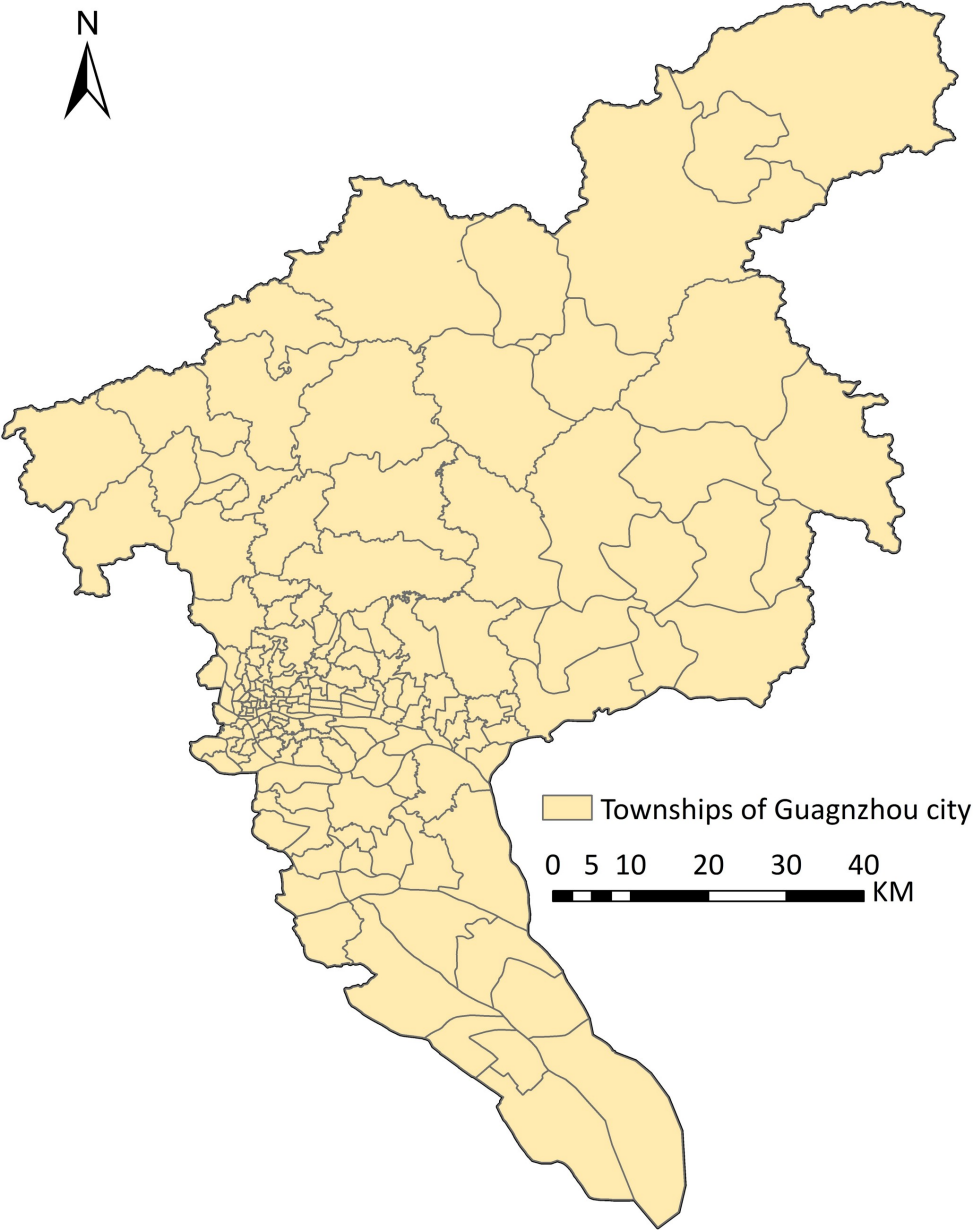
Study area. The 167 townships of Guangzhou City.

Fig 1 shows the 167 townships of Guangzhou, which were used in this study for intra-urban dengue predictions. The geographic data was obtained from Guangdong CDC. Approximately 20% of the townships have areas less than 2km^2^, 37% have less than 5km^2^, and 52% have less than 10km^2^, which is a fine spatial resolution compared to that for existing dengue forecasting studies.

#### Dengue case data

Individual-level dengue cases between January 1, 2015, and September 22, 2019, with residential addresses registered in Guangzhou were obtained from the Guangdong Center for Disease Control and Prevention, which has access to the China National Notifiable Disease Surveillance System. The characteristics of each case include sex, age, nationality, residential address, onset date, diagnosis date, type (imported or local), etc. The coordinates of the cases were obtained from the residential addresses by the geocoding application programming interface provided by Baidu Maps, one of the most popular web mapping, navigation, and location-based service providers in China.

**Fig 2:**
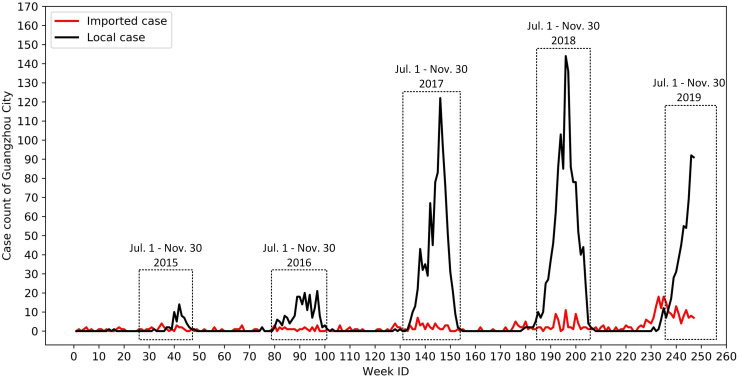
Weekly dengue case count of Guangzhou from January 2015 to September 2019. July 1 to November 30 was determined as the annual outbreak period of Guangzhou in this study.

The collected individual cases were aggregated into a weekly case count for each township based on the onset date during the study period. Fig 2 presents the weekly imported and local case counts of the whole city during the study period, respectively. The number of dengue cases in Guangzhou during the study period is very small as strict intervention measures have been implemented, rendering the dengue prediction task extremely difficult, particularly at a fine spatial resolution. According to Fig 2, we identified July 1 to November 30 as the annual outbreak period of Guangzhou.

#### Meteorological data

**Fig 3:**
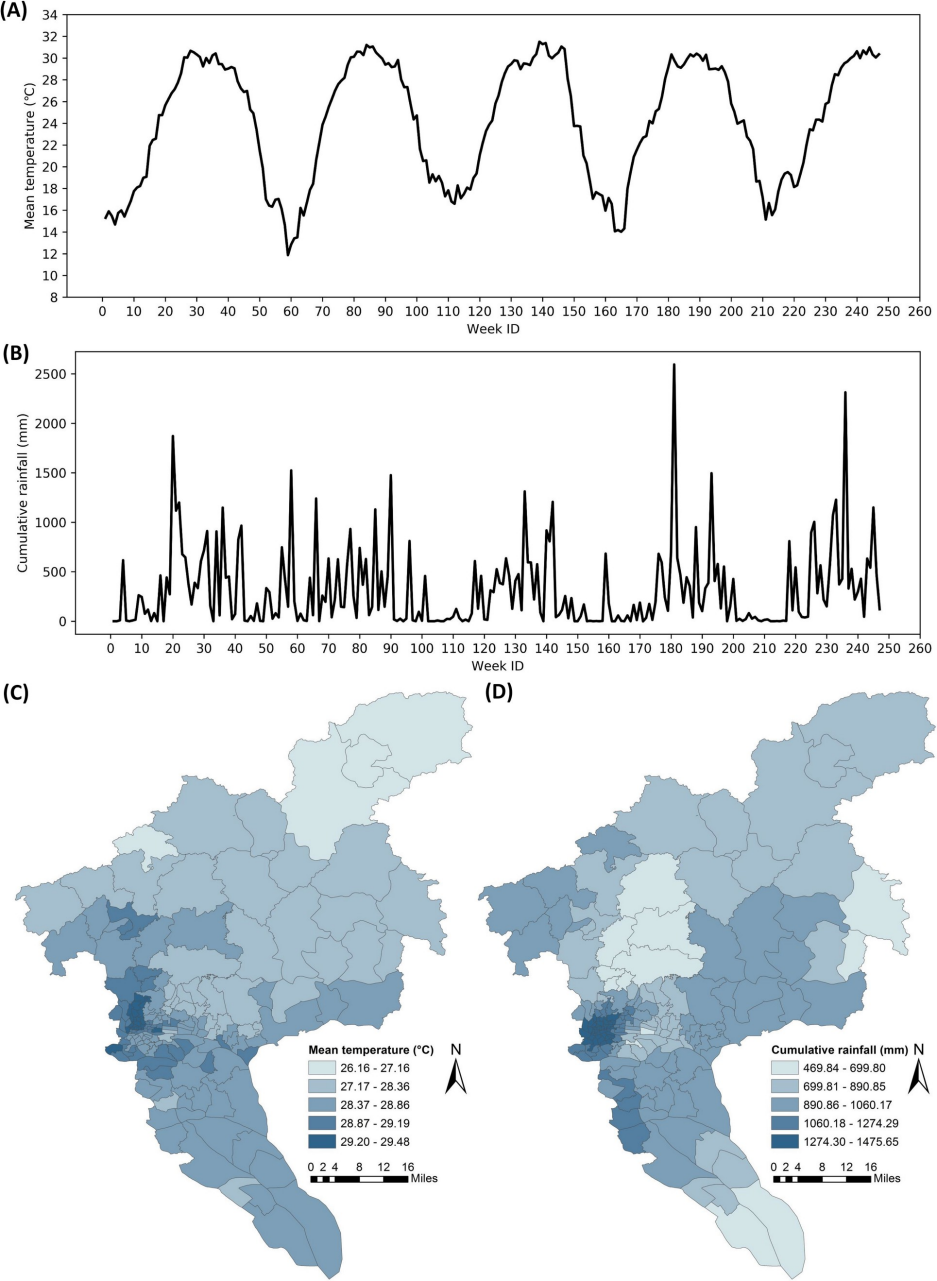
Meteorological data in Guangzhou City. (A) Weekly mean temperature of an arbitrarily selected township from January 2015 to September 2019. (B) Weekly cumulative rainfall of an arbitrarily selected township from January 2015 to September 2019. (C) Weekly mean temperature and (D) weekly cumulative rainfall of all townships within the city during the week of September 12–18, 2016.

The meteorological data, including daily mean temperature and daily rainfall recorded by nearly 300 weather stations in Guangzhou during the study period, were obtained from the Guangdong Meteorological Bureau. The station-based data were spatially interpolated to a fine resolution (500 m) using the ordinary Kriging method where Spherical function was chosen for modeling the empirical semivariogram. Then, the interpolated raster data were averaged (for temperature) or summed (for rainfall) at the township level. Figs 3A and 3B illustrate the weekly mean temperature and cumulative rainfall of one arbitrarily selected township during the study period. Fig 3C and 3D show the weekly mean temperature and cumulative rainfall of all townships within the city during an arbitrarily selected week (i.e., September 12–18, 2016).

#### Population data

The population data of Guangzhou used in this study were obtained from the WorldPop Project (https://www.worldpop.org/) [37,38]. The WordPop datasets have been widely used by researchers and policy makers [29]. The 100-m gridded population (2015) of Guangzhou was aggregated at the township level. A township with larger population implies that there are more hosts for the mosquito vectors and the incidence rate is more likely to be higher.

#### Mobile phone data

The population flows between townships during 1 week of September 2019 were derived from the mobile phone signaling data collected by the China Mobile Telecommunications Company (one of the three major telecommunication operators in China) with a sampling interval of less than one hour. Table 1 presents the records of one cellphone user in the mobile phone data; each record includes the user’s anonymous ID, and the timestamp and coordinate of the cell tower she/he was connected. By aggregating the individual-level records, we can derive the total count of human movements from one township to another during the week, and construct a directed and weighted population interaction network. As human mobility has strong regularities or patterns in both individual and aggregated levels [39–41], we assumed that the relative interaction strengths between townships would not change a lot during the study period, and used the population interaction network of one week as a representative of all weeks.

**Table 1:** Records of one cellphone user in the mobile phone data.

User ID	Timestamp	Longitude	Latitude
0****317af5a17	00:13:44	113.891	22.585
0****317af5a17	01:11:10	113.891	22.585
0****317af5a17	13:10:59	114.083	22.544
0****317af5a17	23:34:57	113.891	22.585

### Introduction of the graph-embedding model Node2Vec

Networks (e.g., transport and social media networks) exist everywhere in both the physical and virtual worlds, making the feature learning of nodes on a graph an emerging task in the field of computer science. In recent years, various graph-embedding algorithms have been proposed to automatically learn high-quality feature vectors from graph structures, which can be used as input for existing machine learning algorithms [42].

**Fig 4:**
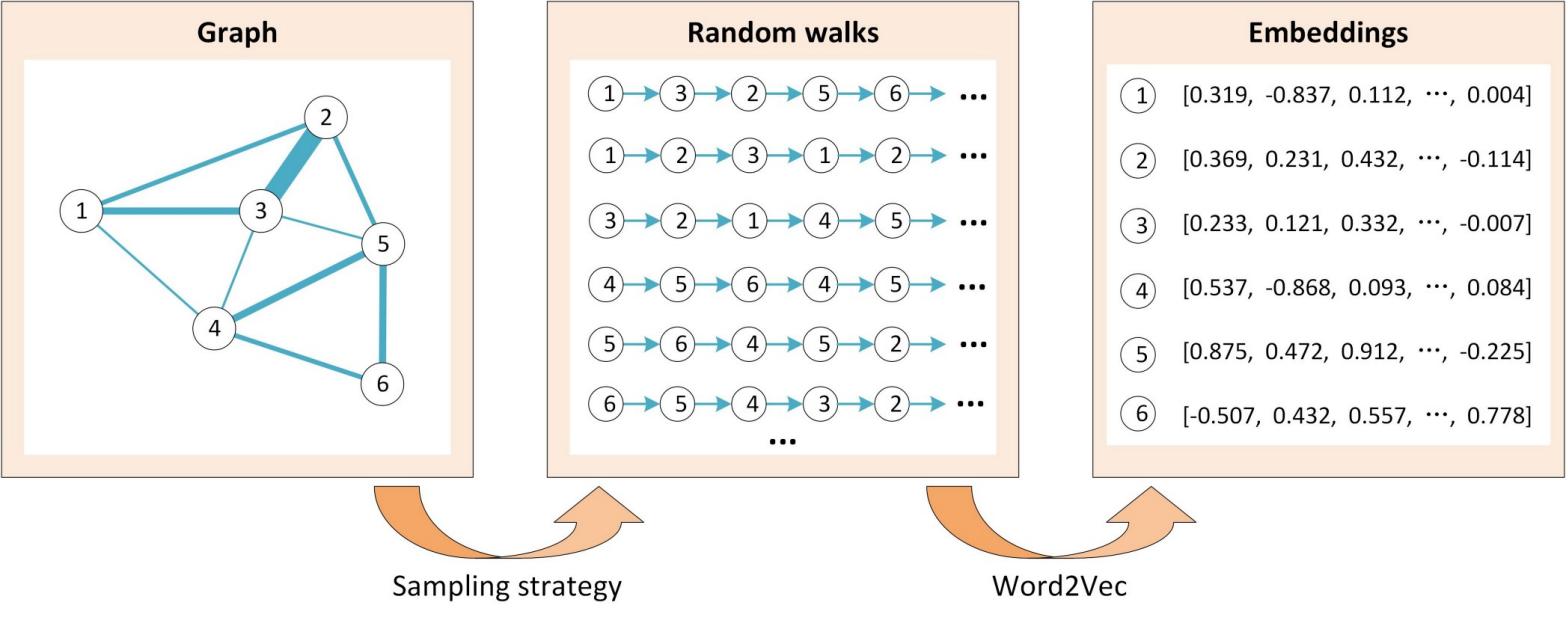
The framework of the Node2Vec model. Based on a certain sampling strategy, many random walks can be generated on the graph. Treating the nodes as “words” and the random walks as “sentences,” the embeddings of the nodes can be learned by feeding these “sentences” into the Word2Vec model.

Node2Vec is one of the most famous graph-embedding algorithms [43], which was developed based on the word-embedding model Word2Vec from the natural language processing domain [44]. Fig 4 presents the framework of the Node2Vec model, which is composed of sampling strategy and Word2Vec. Node2Vec follows the intuition that each node in a graph can be treated as a word, and a random walk on a graph can be treated as a sentence (i.e., word sequence). Then, using the Word2Vec model, the embeddings of the nodes can be automatically learned from their neighborship in the random walks (node sequences). Since nodes with strong interactions (e.g., closely connected with large weight) on the graph would co-occur frequently as neighbors in the random walks, their embeddings would be more similar after training by the Word2Vec model. In this manner, the embeddings of the nodes can serve as meaningful features with graph structure implicitly embedded.

The innovative feature of Node2Vec is that it allows for flexible sampling in generating random walks on the graph to better capture the graph structure. The sampling strategy is related to search bias *a* and edge weights. Assuming that the walk just transitioned from node <*v*_*i*−1_> to node <*v*_*i*_>, the search bias *a* of visiting one of its neighbors <*v*_*i*+1_> in the next step is defined as:

$$\{\begin{array}{ll} \frac{1}{p} & \mathrm{if}\;d\left(v_{i-1},v_{i+1}\right)=0\\ 1 & \mathrm{if}\;d\left(v_{i-1},v_{i+1}\right)=1\\ \frac{1}{q} & \mathrm{if}\;d\left(v_{i-1},v_{i+1}\right)=2 \end{array},$$ (1)

where *d* is the topological distance. If the return parameter *p* is low (<1), it would lead the walk to backtrack a step and this would keep the walk “local” close to the starting node. If the in-out parameter *q* is low (<1), the walk is more inclined to visit nodes that are further away from the initial node.

**Fig 5:**
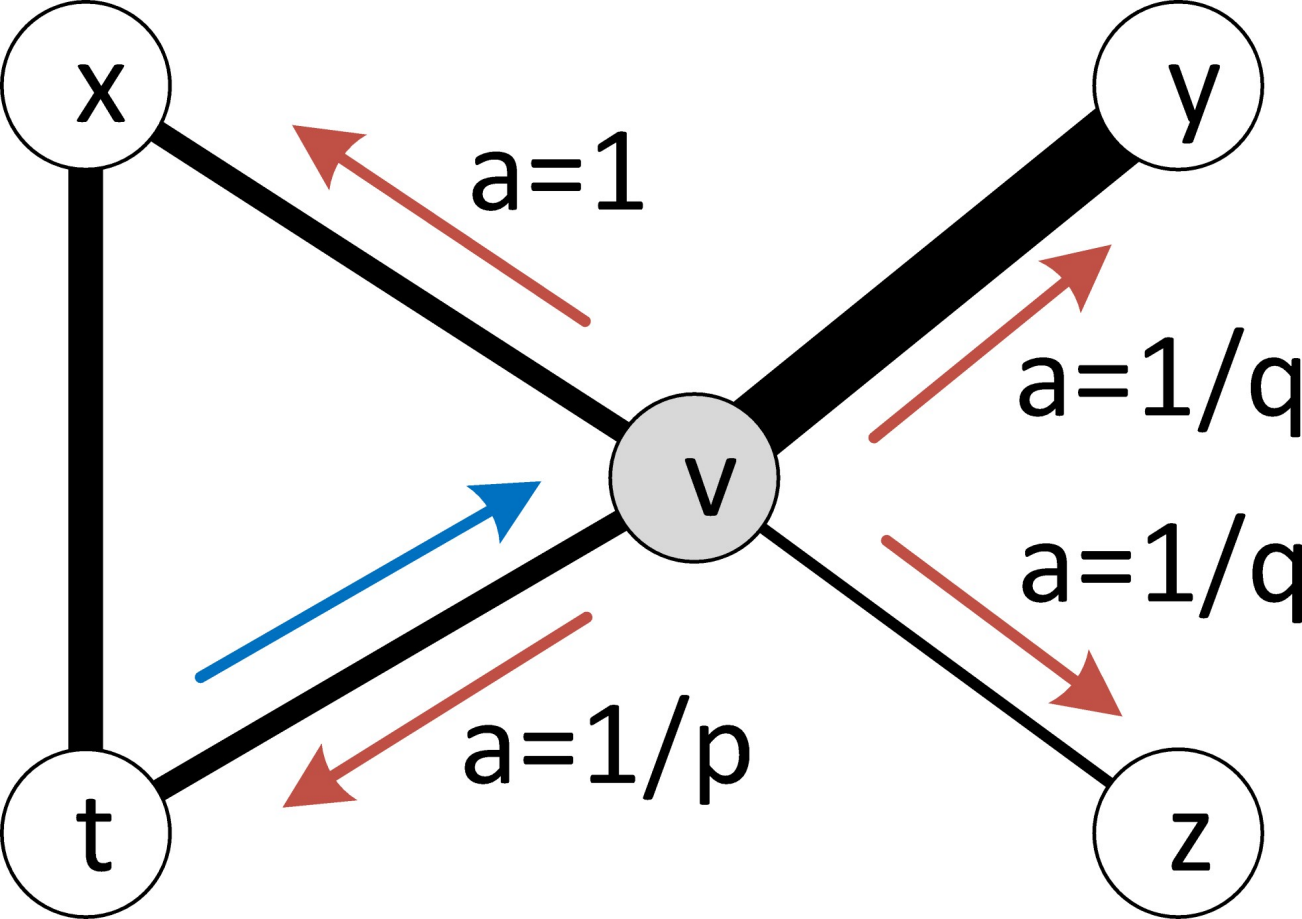
Sampling strategy of random walks in the Node2Vec model. The walk just transitioned from node <*t*> to node <*v*> and is now evaluating its next step out of node <*v*>. The transition probability from <*v*> to any one of its neighbors (i.e., <*t*>, <*x*>, <*y*>, and <*z*>) depends on the search bias *a* and the edge weight between them (thicker lines indicate larger edge weights).

The transition probability from node <*v*> to any one of its neighbors depends on the product of the search bias and the edge weight. In the example shown in Fig 5, the search bias from <*v*> to <*y*> is the same as that from <*v*> to <*z*>; however, as the weight of edge <*v*, *y*> is larger than the weight of edge <*v*, *z*>, the transition probability from <*v*> to <*y*> would be larger than that from <*v*> to <*z*>. Through such sampling strategy, the structure of the graph is implicitly involved in the random walks (node sequences).

The generated random walks in the form of node sequences are then fed into the Word2Vec model as “sentences” to learn the embeddings of the nodes. Word2Vec was initially created to learn the embeddings of words according to the context relation of the words in the corpus that words frequently co-occurring as contexts (e.g., “boat”-“water”) or having similar contexts (e.g., “boat”-“ship”) would have similar embeddings. Similarly, by applying the Word2Vec model to the generated random walks, nodes strongly interacting with each other in the graph would have more similar embeddings and hence can serve as meaningful features.

### Approach for enhancing intra-urban dengue forecasting

**Fig 6:**
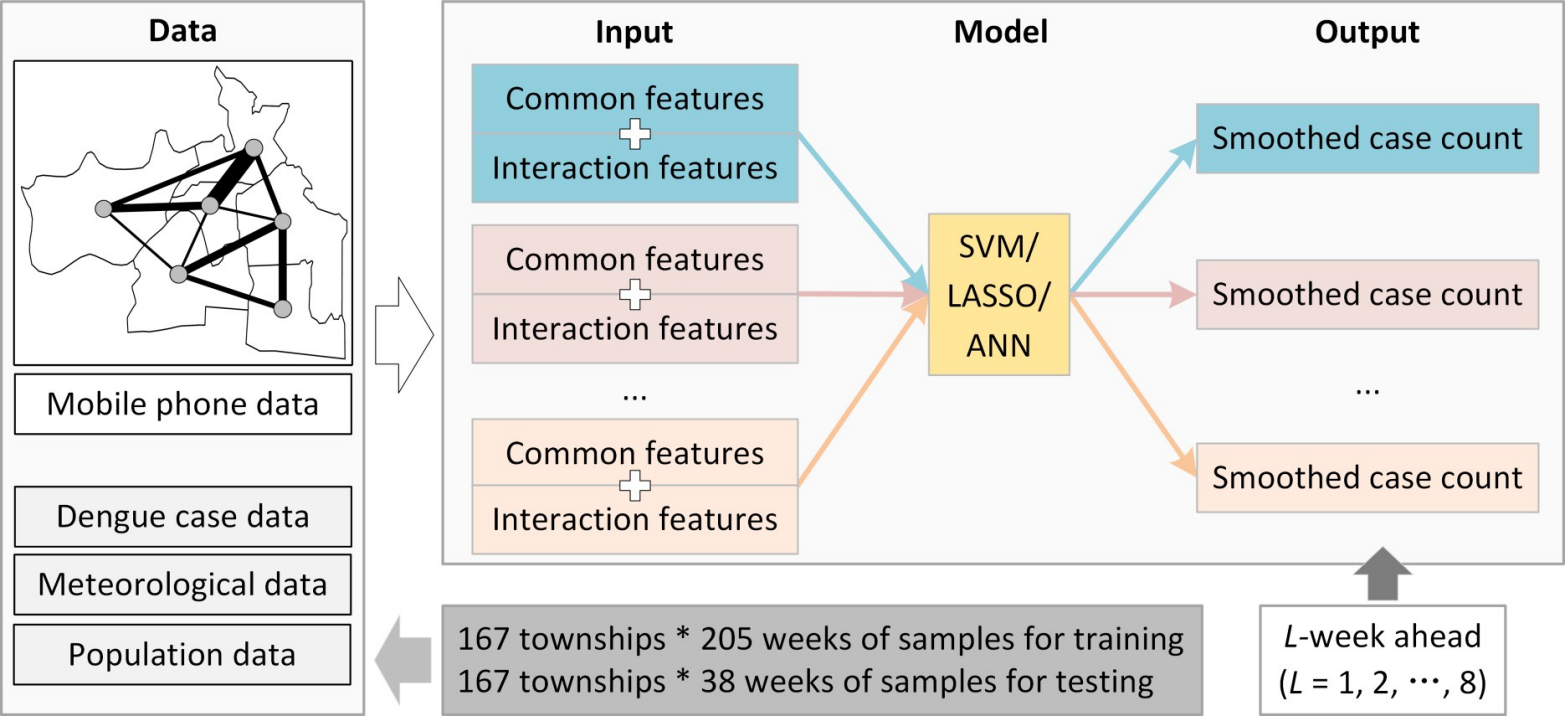
Framework of intra-urban dengue forecasting approach. Common features were extracted from the dengue case data, meteorological data, and population data, while the interaction features were learned from the mobile phone data. The interaction features were combined with the common features to enhance the models (i.e., SVM, LASSO, and ANN) for *L*-week ahead dengue forecasting.

Here, we propose an approach to enhancing the fine-grained intra-urban dengue forecasting by integrating spatial interactions of human movements. In particular, we applied the Node2Vec model to learn the embeddings of townships from the spatial interaction graph constructed from population flows between townships; the learned embeddings were taken as interaction features to enhance the forecasting process together with the commonly used dengue related features. Fig 6 shows the framework of the approach; eight SVM/LASSO/ANN models were separately trained for 1- to 8-week ahead dengue forecasting. The output of the model is derived from the temporal trend of dengue case count in the township, which will be introduced in the following “forecasting model construction” part. Taking Guangzhou as a study case, data collected from January 26, 2015, to December 31, 2018 (a total of 167 townships*205 weeks of samples) were used for training, and data collected from January 1, 2019 to September 22, 2019 (a total of 167 townships*38 weeks of samples) were used for evaluation.

Next, we introduce the processes of interaction feature learning, common feature extraction, forecasting model construction, and forecasting performance evaluation in detail.

#### Interaction feature learning

This study used the graph-embedding model Node2Vec to learn the embeddings of townships by treating each township as a node and the population flow from one township to another as weight of the directed edge. As we introduced previously, search biases and edge weights determine the method of random walks for capturing the graph structure. In order to avoid the walks being trapped in local structure, here we set the return parameter *p* to a large value (*p* = 4), and the in-out parameter *q* to a small value (*p* = 0.025). Then, the generated random walks were fed into the Word2Vec model to derive the embeddings of townships in the form of *N*-dimensional real-valued vectors (*N* = 64 was commonly used). Through this way, strongly interacted townships would have more similar embeddings, which can serve as interaction features to enhance dengue forecasting.

#### Common feature extraction

This study used epidemical, meteorological, and sociodemographic variables as common features, which have been widely used and proven as important predictors for dengue forecasting. As shown in Table 2, 11 common features were extracted for each township from the past cases (including imported and local cases), mean temperature, cumulative rainfall, and population; the first three types of features are spatio-temporal variables with time lags up to 4 weeks, while the last one is a spatial variable.

**Table 2:** Common features extracted for each township.

No.	Category	Feature
1	Epidemical	Weekly case count (lag 1)
2		Weekly case count (lag 2)
3		Weekly case count (lag 3)
4		Weekly case count (lag 4)
5		Cumulative case count of past 4 weeks
6	Meteorological	Weekly mean temperature (lag 1)
7		Weekly mean temperature (lag 2)
8		Weekly mean temperature (lag 3)
9		Weekly mean temperature (lag 4)
10		Cumulative rainfall of past 4 weeks
11	Sociodemographic	Population

#### Forecasting model construction

This study selected SVM, LASSO, and ANN as basic dengue forecasting models, which have been widely used and proven effective in existing literature. Specifically, we used a linear kernel in the SVM-based regression model, and set *alpha* (i.e., the constant that multiplies the L1 term in the optimization objective) as 0.001 in the LASSO-based regression model. As for the ANN-based regression model, we used a Multi-layer Perceptron regressor with one hidden layer of 100 neurons, applied “tanh” as the activation function, and set *learning rate* as 0.001. The maximum number of iterations was set to a large value (i.e., 3000) for all the three models. Those models were all implemented using the machine-learning Python package “scikit-learn” [45–47]. Parameters that not mentioned above were applied with the default values given by the package.

**Fig 7:**
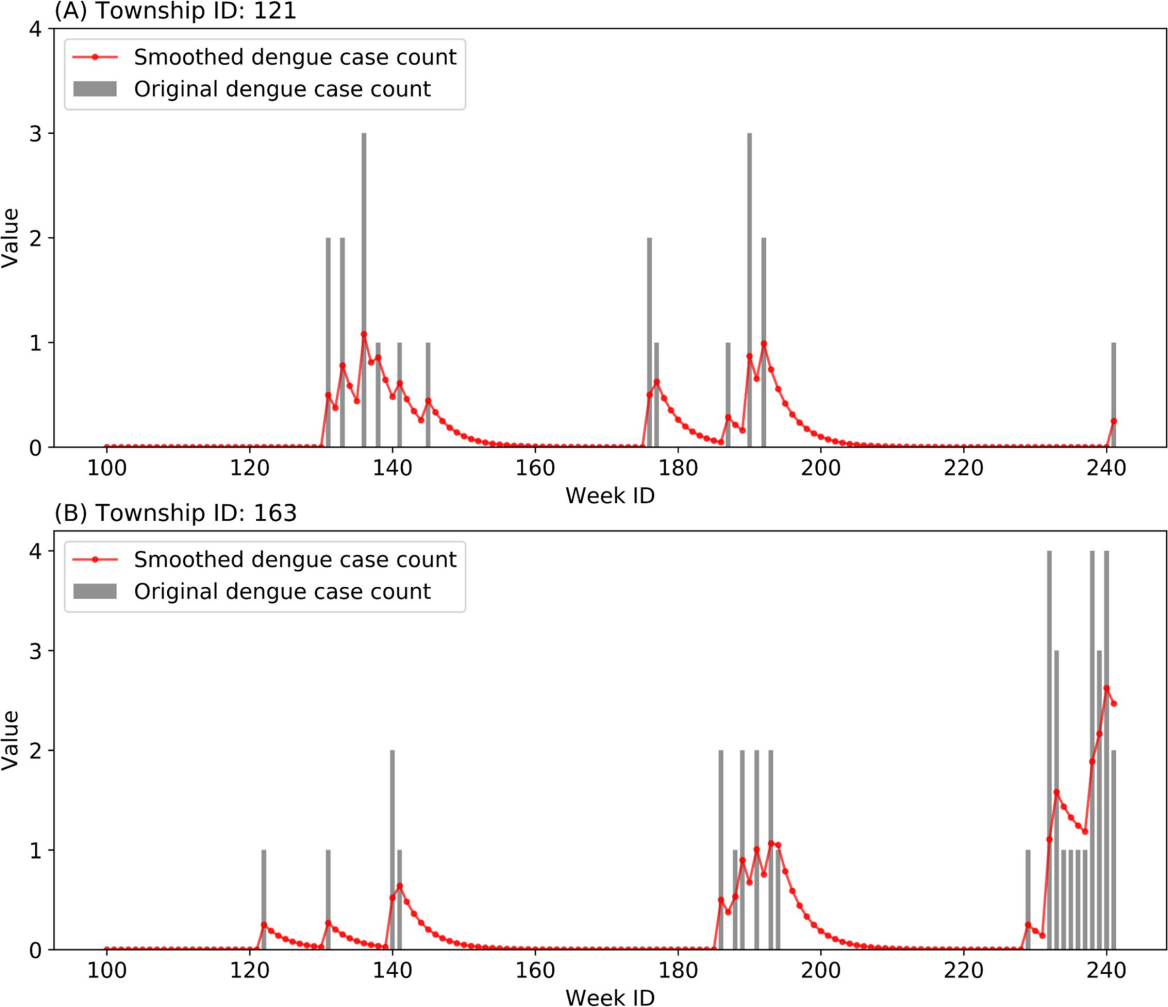
Exponential smoothing (*α*_*s*_ = 0.25) applied to the time series of weekly dengue case count of two townships.

The inputs of the models consisted of 64-dimensional interaction features and 11-dimensional common features. Each dimension of the feature vector was normalized to a range between 0 and 1 using the Min-Max feature scaling method. As for the outputs, because of the fact that dengue cases at the township level were very few in Guangzhou during the study period, we applied an exponential smoothing technique to the time series of the weekly local case count in each township, and took the smoothed value as the output of the forecasting model. Denoting the raw time series as {*x*_*t*_}, the smoothed time series {*s*_*t*_} was obtained by the following formulas:

$$s_{0}=x_{0},$$ (2)

$$s_{t}=\alpha_{s}x_{t}+(1-\alpha_{s})s_{t-1},t>0,$$ (3)

where *α*_*s*_ is the smoothing factor in the range of [0, 1]. Setting *α*_*s*_ as 0.25, we derive the smoothed dengue case count of each township in each week as the output of the forecasting models. Taking two randomly selected townships as examples, the time series of weekly case counts during a period before and after data smoothing are displayed in Fig 7. The data-smoothing scheme can help retain the latent temporal patterns of dengue epidemic, and mitigate the unknown and uncertain influence of human intervention in various townships.

#### Forecasting performance evaluation

The aim of dengue forecasting conducted at a large spatial scale (e.g., country, state/province, and city) is to provide an early warning, while a fine-grained intra-urban forecasting focuses more on identifying regions with relatively higher risk in the near future, which can facilitate precise prevention and control despite limited resources. Therefore, besides measuring the Pearson correlation coefficient of the predicted and observed result (i.e., the smoothed dengue case count), we also propose to assess the forecasting performance from a spatial perspective by evaluating the accuracy of their identification of high-risk regions.

In particular, a “hit rate” metric was used to measure the proportion of dengue cases captured by the top *m*% predicted high-risk townships. Specifically, for a specific epidemiological week, sorting the 167 townships by their predicted smoothed case counts from high to low, the top m% high-risk townships indicate the top 167* m% townships in the rank sequence. The hit rate metric of week *t* can be calculated as:

$${Hit\;rate}_{t}=\frac{N_{m,t}}{N_{t}},$$ (4)

where *N*_*m*,*t*_ presents the number of observed cases inside the top *m*% predicted high-risk townships and *N*_*t*_ denotes the total number of observed cases within the city. A high hit rate indicates that high-risk townships during the week have been well identified by the forecasting model. For each forecast window, the hit rates of all prediction weeks in the validation dataset were averaged.

## Results

In this part, we first demonstrated the forecasting results from temporal and spatial perspectives, and then evaluated the performance of the proposed approach quantitatively using the Pearson correlation coefficient and hit rate metric.

### Temporal and spatial demonstrations of forecasting results

**Fig 8:**
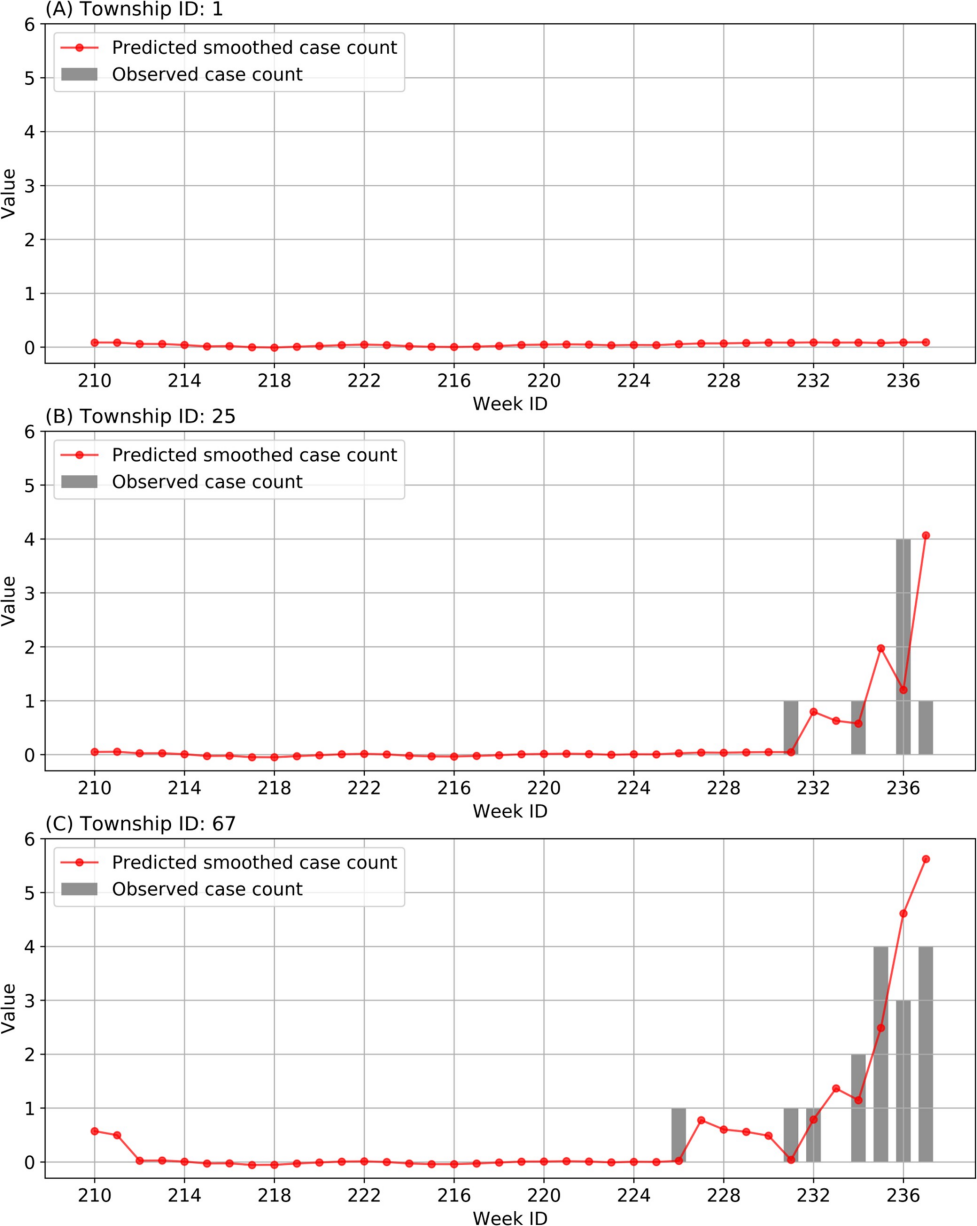
Predicted smoothed case counts and observed case counts of three randomly selected townships in Guangzhou during the validation period. The smoothed case counts were predicted by the 1-week ahead SVM-based model using both common features and interaction features.

Fig 8 presents the smoothed case counts of three townships in Guangzhou predicted by the 1-week ahead SVM-based model using both common features and interaction features. It indicates that our predicted result at the township level is generally in parallel with the temporal trend of the dengue epidemic in reality, which can serve as an early warning for preparing prevention and control measures.

**Fig 9:**
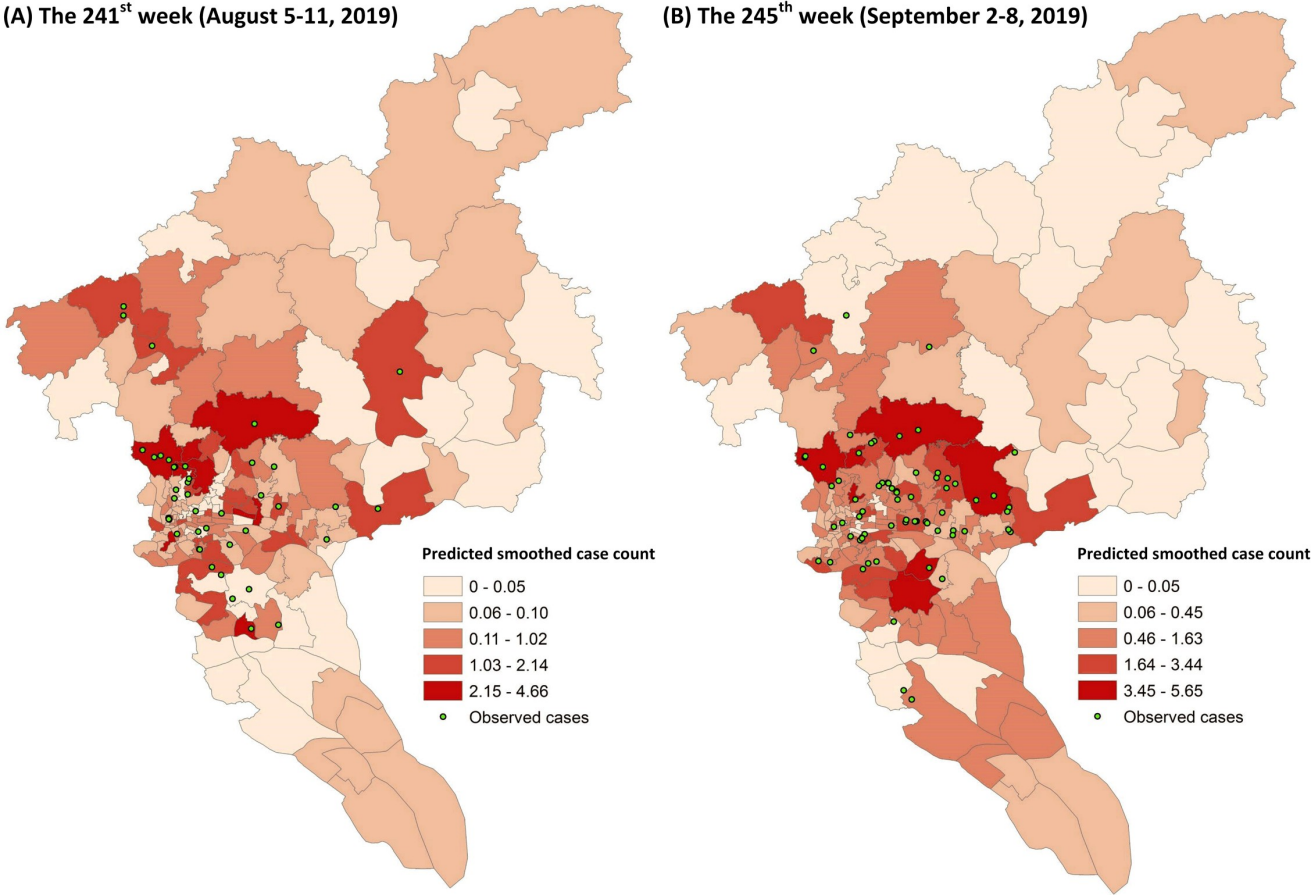
Predicted smoothed case counts of all townships during two different weeks. The smoothed case counts were predicted by the 1-week ahead SVM-based model using both common features and interaction features.

Fig 9 demonstrates the predicted smoothed case counts of all townships across the city during two different weeks (i.e., the 241^st^ week and the 245^th^ week). According to the statistic result, the top 30% and 50% high-risk townships (i.e., the top 50 and 84 high-risk townships) can capture 63.2% and 81.6% of the actual dengue cases during the 241^st^ week, respectively. Whereas for the 245^th^ week, 72.5% and 89.9% of the actual dengue cases can be captured by the top 30% and 50% high-risk townships, respectively. This indicates that the high-risk townships can be generally identified by the proposed approach for conducting prevention and control measures.

### Performance comparison and evaluation

**Fig 10:**
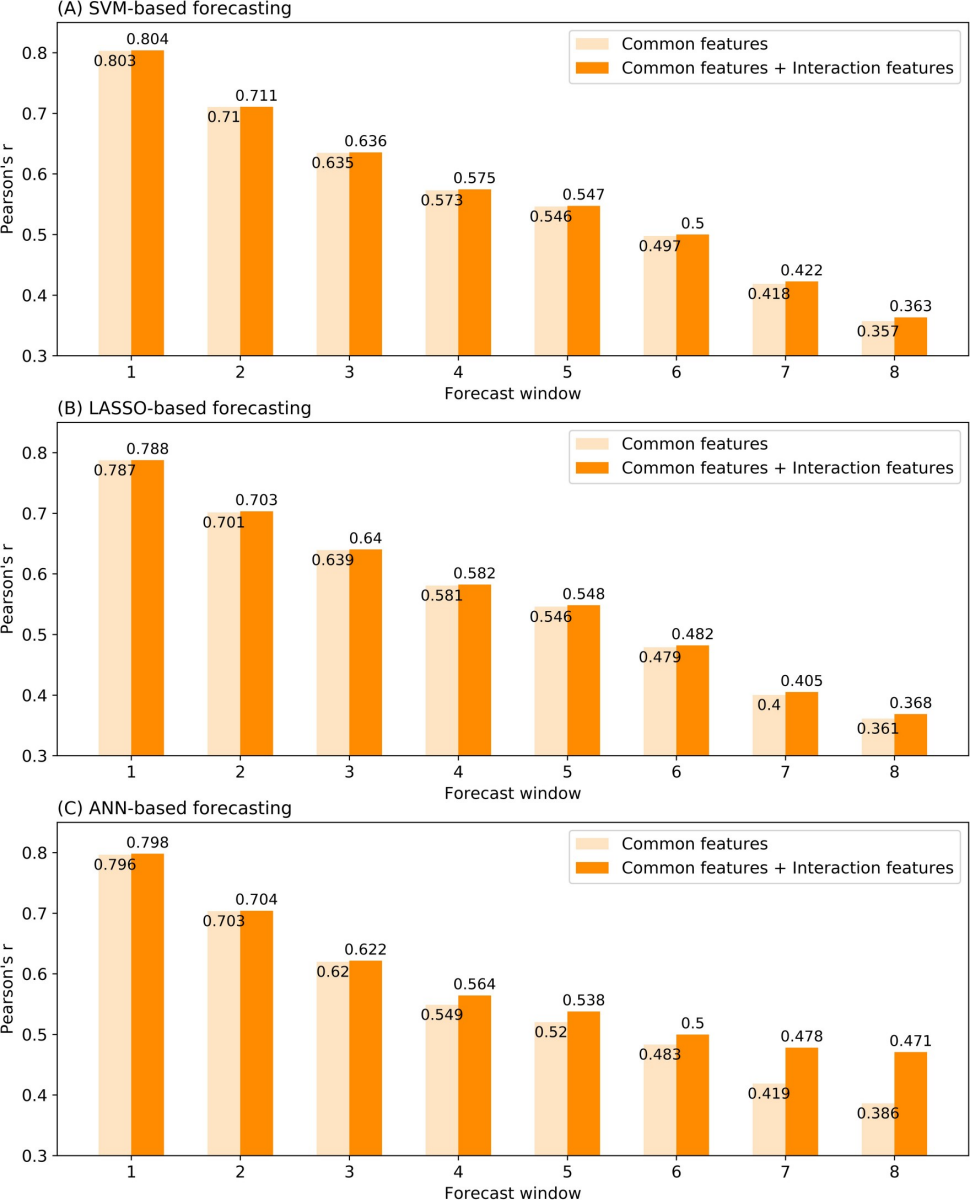
Performance comparison of models with and without interaction features based on the Pearson correlation coefficient of predicted and actual smoothed case counts. (A) SVM-based forecasting. (B) LASSO-based forecasting. (C) ANN-based forecasting.

First, the Pearson correlation coefficient was applied to measure the predicted and actual smoothed case counts of samples in the validation dataset. As shown in Fig 10, Pearson’s r gradually decreased with the increase of the forecast window, and all the three types of models with combined interaction features outperform those with only common features, indicating the usefulness of the interaction features.

**Fig 11:**
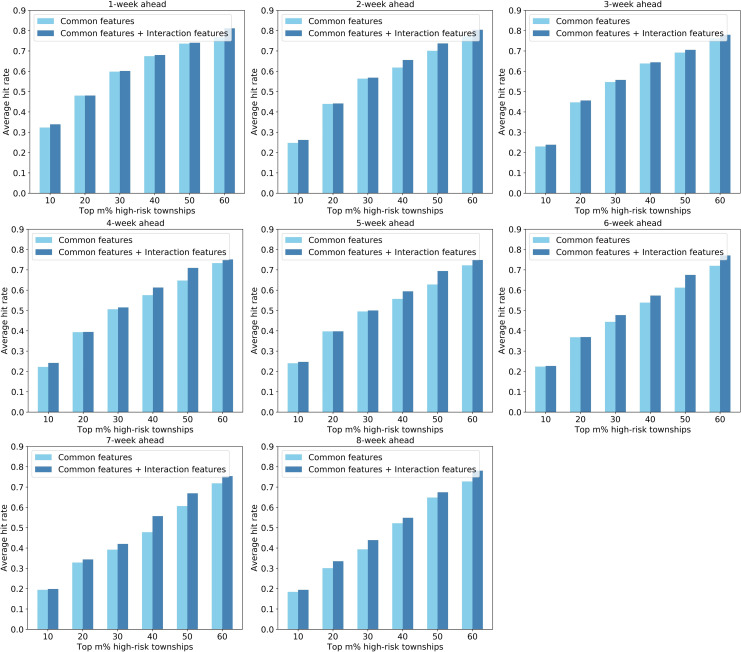
Performance comparison of the SVM-based models with and without interaction features based on the hit rate metric.

**Fig 12:**
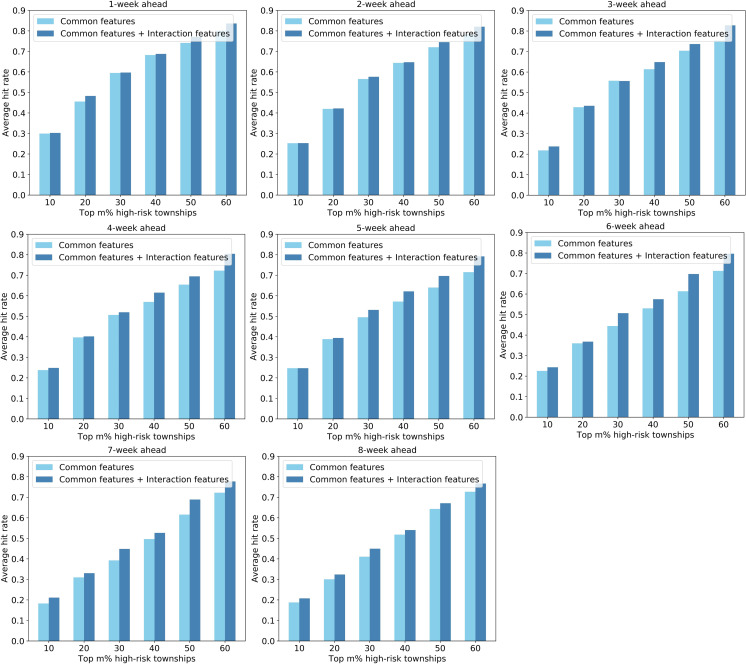
Performance comparison of the LASSO-based models with and without interaction features based on the hit rate metric.

**Fig 13:**
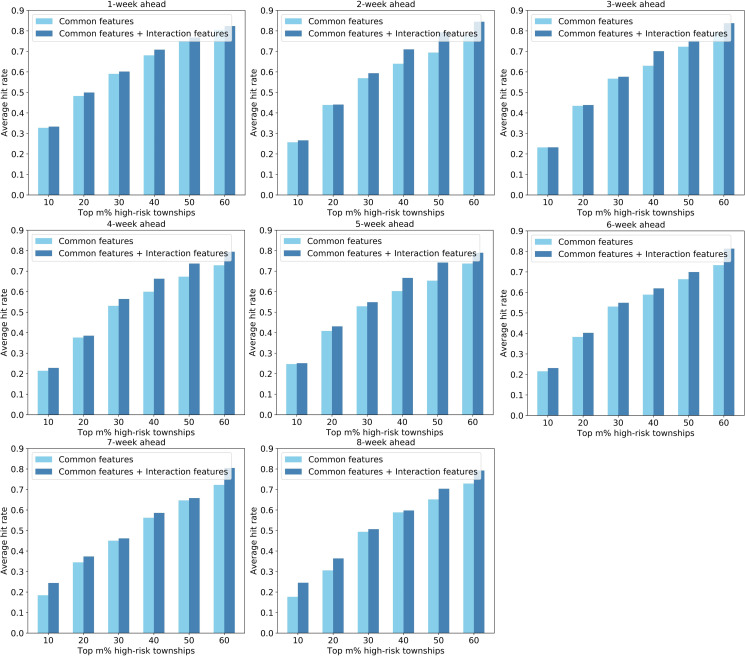
Performance comparison of the ANN-based models with and without interaction features based on the hit rate metric.

Second, the hit rate metric was used to measure the ability of the models to identify high-risk townships in advance. Fig 11, Fig 12, and Fig 13 shows the average hit rates during the outbreak period derived from the SVM-, LASSO-, and ANN-based models, respectively. As for the 1-week ahead forecasting, the predicted top 30% high-risk townships (i.e., top 50 high-risk townships) can capture about 60% of the dengue cases across the city. When conducting 8-week ahead forecasting, the top 30% high-risk townships can capture about 50% of the dengue cases in the city. Even though not obvious as the Pearson correlation coefficient, the average hit rate generally decrease with the increase of forecast window. More importantly, the results indicate that models using both common and interaction features as inputs generally perform better than that using only common features, further verifying that our strategy of integrating spatial interactions of human movements is effective in enhancing dengue forecasting.

## Discussions

Highly dynamic population flows within a city complicate and accelerate dengue virus transmission, increasing the likelihood of large and uncontrollable disease outbreaks in urban areas. For this reason, accurately forecasting the spatial distribution of dengue cases within a city is important for government agencies to establish early and targeted prevention and control. While on the other hand, even though researchers have realized the importance of human mobility in virus transmission [26–29], the real population movement data have not been well utilized in dengue forecasting.

This study proposes a framework for enhancing fine-grained intra-urban dengue forecasting by integrating spatial interactions of human movements between urban regions. Treating intra-urban regions as nodes and the population flows between them as edge weights, we applied a word-embedding model called Node2Vec to learn the embeddings of the regions from their population interaction network. The learned embeddings can serve as interaction features to enhance intra-urban dengue forecasting. Through a case study conducted in Guangzhou City, we found that forecasting models employing both interaction features and the commonly used features achieved better performance than those using common features alone, proving the effectiveness of our strategy for incorporating spatial interactions of human movements within the city.

The highlights of this study can be summarized as follows.

1. While current dengue predictions have been primarily conducted at the national, subnational, and city levels to flag an outbreak, this study focused on the fine-scale intra-urban environment and was able to identify high-risk intra-urban regions for precise dengue prevention and control.
2. We proposed a novel strategy for integrating spatial interactions of human movements by introducing the graph-embedding technique to learn embeddings of urban regions from their population interaction network. The learned embeddings were proved to be useful interaction features for enhancing intra-urban dengue forecasting. Actually, population flow are a kind of relational data usually represented as <*x*_1_,*x*_2_,*s*>, where *x*_1_ and *x*_2_ are the origin and destination, and *s* is the interaction strength between them [39]. The introduced graph-embedding technique transfers the relational data into the form of <*x*,*a*>, where *a* is the attribute of location *x*. Through this way, the human mobility data can be more conveniently applied in forecasting models of dengue fever and other infectious diseases.
3. We evaluated dengue forecasting performance from a spatial perspective by using the hit rate metric, which is in parallel with the aim of identifying high-risk regions within the city for precise prevention and control. In addition, compared to other commonly used metrics such as AUC (i.e., area under the receiver operating characteristic curve), the physical meaning of hit rate metric is more intuitive and understandable for the staff of CDC in prevention and control practice.

However, our research has some limitations. First, the dengue case data used in this study are dependent on notifiable data, but there is a possibility that mild or asymptomatic cases may not be diagnosed and reported. Second, even though integrating interaction features enhanced the performance of the forecasting models, the improvements were not very significant in our study case. Considering the dengue cases at the township level of the study area are sparsely distributed, which generates a highly challenging spatiotemporal prediction task, this result is reasonable. However, since our proposed approach has achieved better performance on such sparse dataset and a difficult prediction task, it can behave better in cities or situations with more densely distributed dengue cases theoretically. Third, as only 1 week of mobile phone positioning data were used in this study, our extracted interaction features are rendered static. If long-term human mobility data would be available in the future, dynamic interaction features can also be trained to better capture the structure of population flow network and enhance dengue forecasting.
